# Persistence of Antibodies against Measles, Mumps, and Rubella after the Two-Dose MMR Vaccination: A 7-Year Follow-Up Study

**DOI:** 10.3390/vaccines12070744

**Published:** 2024-07-05

**Authors:** Nasiri Sarawanangkoor, Nasamon Wanlapakorn, Donchida Srimuan, Thaksaporn Thatsanathorn, Thanunrat Thongmee, Yong Poovorawan

**Affiliations:** 1 Center of Excellence in Clinical Virology, Department of Pediatrics, Faculty of Medicine, Chulalongkorn University, Bangkok 10330, Thailand; nasiri.s001@gmail.com (N.S.); nasamon.w@chula.ac.th (N.W.); donchida.s@gmail.com (D.S.); thaksapohnl@hotmail.com (T.T.); tata033@hotmail.com (T.T.); 2 The Royal Society of Thailand, Dusit, Bangkok 10300, Thailand

**Keywords:** measles, mumps, rubella, antibody, persistence, children

## Abstract

In 2014, the Expanded Program on Immunization of Thailand changed the timing of the second dose of the measles–mumps–rubella (MMR) vaccine from 4–6 years to 2.5 years, while maintaining the first dose at 9 months of age. This study aimed to examine the dynamics and durability of immune responses induced by the two-dose MMR vaccine in a group of 169 Thai children from 4 to 7 years of age (4.5 years after the second MMR dose). We followed a cohort of healthy children from a clinical trial (ClinicalTrials.gov NCT02408926) where they were administered either the Priorix vaccine (GlaxoSmithKline Biologicals, Rixensart, Belgium) or M-M-RII (Merck & Co., Kenilworth, NJ, USA) at 9 months and 2.5 years of age. Blood samples were collected annually from ages 4 to 7 years. Anti-measles, -mumps, and -rubella IgG levels were evaluated using the enzyme-linked immunosorbent assay (EUROIMMUN, Lubeck, Germany). A total of 169 children completed this study. Over the 4.5 years following the two-dose MMR vaccination, we observed a decline in the seroprotection rates against measles and mumps, but not rubella. Longitudinal monitoring of antibody persistence, among other strategies, will help predict population-level immunity and inform public health interventions to address potential future outbreaks.

## 1. Introduction

Measles commonly manifests with symptoms such as high-grade fever, cough, and conjunctivitis; however, it can also result in severe complications, including pneumonia, encephalitis, and subacute sclerosing panencephalitis [1]. These severe outcomes predominantly affect young children [2]. As a highly contagious airborne-transmitted disease, measles is a significant threat to public health. The live attenuated measles vaccine, especially when administered in a two-dose regimen, demonstrates high protective efficacy. This underscores the importance of maintaining high population immunity with at least 95% vaccination coverage with two doses of the measles vaccine [3]. This measure is essential for minimizing morbidity and mortality associated with measles and working towards the goal of measles eradication. According to the Immunization Agenda 2030 (IA2030), stronger health systems and increased measles vaccination coverage resulted in a 73% global decrease in measles-associated mortality between 2008 and 2018 [4]. However, regional elimination has not been sustained, and there has been a resurgence in measles cases and deaths globally, with cross-border importations and immunity gaps leading to large outbreaks. Globally, coverage for the first measles-containing vaccine dose has plateaued at around 85% over the past decade, and the second dose coverage has increased to 69%, which is insufficient. To address this, supplementary vaccine delivery methods, including planned campaigns and routine immunization intensification, are necessary. Ensuring every child receives two timely doses of measles-containing vaccine and maintaining effective measles surveillance are critical indicators of a strong immunization program. Measles cases highlight gaps in population immunity, indicating inadequate access or uptake of vaccines. Thus, a robust immunization program is crucial to responding to this challenge. Additionally, the Measles and Rubella Strategic Framework (MRSF) 2021–2030 was developed to provide high-level guidance for formulating regional and national strategies and operational plans to achieve “a world free from measles and rubella”. [5] Effective implementation of these strategies is vital for sustaining progress and achieving long-term eradication goals.

The timing of the first and second doses of the measles-containing vaccine (MCV1 and MCV2) varies globally due to differences in measles transmission rates, health service infrastructure, and the ability to reach children at different ages. In the USA and many countries in Europe, the MCV1 is given between 12 and 19 months and the MCV2 at school entry (4–6 years) [6,7]. This schedule is suitable for countries where measles transmission and the risk to infants is low. In countries with ongoing measles transmission and high infant mortality risk, MCV1 should be administered at 9 months of age to provide protection during infancy. On-time delivery of MCV1 is crucial in these settings. Additionally, the World Health Organization recommended that MCV2 should be given routinely at 15–18 months, with a minimum interval of 4 weeks between MCV1 and MCV2 [8]. Administering MCV2 in the second year of life helps immunize those who missed or did not respond to MCV1, reduces the accumulation of susceptible children, and decreases the risk of outbreaks. A phase III vaccine trial in India found that administering MCV1 at 9 months, and MCV2 at 15 months of age induced a robust immune response to measles, mumps, and rubella, with acceptable safety profiles [9].

Since Thailand is a country where ongoing measles transmission and the risk of measles mortality remain high, the MCV1 is administered at 9 months of age [10]. According to the Thai national vaccination policy, administration of MCV2 was moved from 4–6 years to 2.5 years in 2014 [11], and then further adjusted to 15–18 months in 2020. This change aligns with the WHO recommendation for high-transmission countries. Additionally, the new schedule (MCV2 at 15–18 months) corresponds to other routine immunizations in Thailand, such as the diphtheria–tetanus–pertussis (DTP) booster and the optional pneumococcal vaccine booster, which can result in improved compliance and coverage. The national vaccine coverage in Thailand for MCV1 reached > 95% between 2011 and 2021 but slightly decreased to 84–86% in 2022–2023 during the COVID-19 pandemic [12]. On the contrary, the national vaccine coverage in Thailand for MCV2 was slightly lower (approximately 85–86%). Measles sporadically persists in Thailand, primarily in areas with low vaccination coverage [13,14]. The potential reasons for pockets of low vaccination coverage and refusal of measles vaccine in Thailand include concerns about vaccine side effects, lack of universal health coverage, religious compatibility of vaccines, and inconsistencies between health services and community lifestyle [15,16].

Alongside, measles, mumps, and rubella vaccinations were combined to form the measles–mumps–rubella (MMR) vaccine. In Thailand, the two-dose mumps vaccination, administered as an MMR vaccine at 9 months and 4–6 years, was implemented in 2010 [17]. This second dose of mumps vaccine was changed to 2.5 years and 18 months in 2014 and 2020, respectively. A previous study in China showed that mumps could occur in school students who had previously received two doses, but not three doses, and that there was an increase in mumps infection risk in both the one-dose and two-dose groups when the time since the last vaccination dose was more than 5 years [18]. These findings suggest a need for further investigation into the necessity of a booster dose of the mumps-containing vaccine for young adults.

Rubella, which manifests as generalized lymphadenopathy, mild fever, and rash, can easily be underdiagnosed and underreported. When the rubella virus infects the fetus, particularly during the first trimester, it leads to miscarriage or congenital rubella syndrome (CRS) [19]. The rubella vaccine was developed to prevent CRS and has been administered as a two-dose regimen in Thailand since 2010. In a previous cross-sectional seroprevalence study among Thai children and adolescents, it was found that a two-dose MMR vaccination resulted in a high rate of rubella seropositivity, although the antibody levels and seropositivity rate declined over time [20].

Our previous study demonstrated the robust antibody response to measles, mumps, and rubella viruses in Thai children following a two-dose MMR vaccine administered at 9 months and 2.5 years [11]. Building on these findings, this present study investigated the long-term persistence of antibodies to measles, mumps, and rubella viruses up to the age of 7 years, or 4.5 years after the second dose of MMR vaccine. The goal of this study was to assess the dynamics and durability of vaccine-induced immune responses to measles, mumps, and rubella following the two-dose vaccination.

## 2. Materials and Methods

### 2.1. Study Design

This study was an extension study providing long-term follow-up of children who had participated in a previous trial (ClinicalTrials.gov NCT02408926) [21]. The present study was an observational descriptive study aimed at obtaining real-world data on antibody dynamics and durability in Thai children following immunization with the two-dose MMR vaccine administered at 9 months and 2.5 years of age. In the previous trial, 315 infants born to mothers who had received a tetanus–diphtheria–acellular pertussis (Tdap) vaccination during pregnancy were randomized to receive either a diphtheria–tetanus–acellular pertussis–hepatitis B–inactivated polio–*Hemophilus influenzae* type b (hexavalent) vaccine (hereafter referred to as the hexavalent group) or a diphtheria–tetanus–whole-cell pertussis–hepatitis B–*Hemophilus influenzae* type b (pentavalent) vaccine (hereafter referred to as the pentavalent group) for primary and first booster vaccination. Details of the randomized design, population, and study objectives have been reported elsewhere [21]. The recent change in the national vaccination program in the timing of the second MMR vaccine provided an opportunity to assess the dynamics of antibody responses to the revised MMR vaccine described in the present study. Children from both the hexavalent and pentavalent groups received two doses of MMR vaccine at 9 months and 2.5 years, as previously described [11]. Data on the dynamics of antibodies to measles, mumps, and rubella viruses from birth to 3 years of age (6 months after receipt of the second MMR vaccine) were previously published [11].

All eligible children in the previous trial were contacted for participation in this extension study which followed the children until 7 years of age. Exclusion criteria included acquired immunodeficiency disorders, chronic illness that could interfere with the completion of the trial, receipt of immunosuppressive therapy, receipt of blood or blood products before or during the study, hypersensitivity to the vaccine used in this trial, and bleeding disorders in which intramuscular injections were contraindicated.

This long-term follow-up study was conducted between May 2019 and November 2023 at the Clinical Research Unit, Center of Excellence in Clinical Virology, Department of Pediatrics, Faculty of Medicine, Chulalongkorn University, Bangkok, Thailand. This study was approved by the Institutional Review Board of the Faculty of Medicine of Chulalongkorn University (IRB no.315/61 and no. 173/63) and was registered with the Thai Clinical Trial Registry (TCTR20231017001).

### 2.2. Study Vaccines and Blood Sampling

The hexavalent and pentavalent groups received the MMR vaccine (Priorix; GlaxoSmithKline Biologicals, Rixensart, Belgium) or M-M-R II (Merck & Co., Kenilworth, NJ, USA) at 9 and 30 months of age. The components of the vaccine and the number of children receiving Priorix and M-M-R II were previously reported [11]. In this study, children were scheduled for blood sampling visits annually from 4 to 7 years of age.

Venous blood samples (6 mL) from all participants were collected in tubes devoid of anticoagulant or preservative agents. Afterward, the samples underwent centrifugation at 1500× *g* (4 °C) for 10 min. The resulting sera were separated and stored at −20 °C until laboratory analysis.

### 2.3. Laboratory Testing

The concentrations of IgG against measles, mumps, and rubella viruses were evaluated using the enzyme-linked immunosorbent assay (EUROIMMUN, Lubeck, Germany). Serum samples were initially diluted 1:101, and further dilutions were performed to yield results within the detection range, according to the manufacturer’s instructions. Anti-measles, -mumps, and -rubella IgG concentrations are expressed in international units (IU)/L, relative units (RU)/mL, and IU/mL, respectively. Anti-measles IgG was divided into five groups: <200 IU/L, 200 to <275 IU/L, 275 to <1000 IU/L, 1000 to <5000 IU/L, and ≥5000 IU/L. Anti-mumps IgG was divided into four groups: <45 RU/mL, 45 to <100 RU/mL, and ≥100 RU/mL. Anti-rubella IgG was divided into four groups: <10 IU/mL, 10 to <50 IU/mL, 50 to <200 IU/mL, and ≥200 IU/mL. The threshold levels for protective immunity, known as the seroprotection rate, are ≥200 IU/L, ≥45 RU/mL, and ≥10 IU/mL for measles, mumps, and rubella, respectively [22]. Antibody levels below the lower limit of quantification (LLOQ) observed in some samples were calculated as half the LLOQ (25 IU/L for anti-measles IgG, 1 RU/mL for anti-mumps IgG, and 0.5 IU/mL for anti-rubella IgG).

### 2.4. Statistical Analysis

Antibody levels are presented as geometric mean concentrations (GMCs) with 95% confidence intervals (CIs). GMCs were calculated by taking the arithmetic means of the log-transformed values of the antibody levels and then converting them to the real value (GMC) using a table of antilogarithms. Comparisons of the antibody levels between hexavalent and pentavalent groups were conducted using the Kruskal–Wallis test with Dunn’s post hoc correction for multiple comparisons at each timepoint. This analysis was conducted on the log-transformed data, which were found to be non-normally distributed. Changes in GMCs of anti-measles, anti-mumps, and anti-rubella IgG at month 84 versus month 36 (6 months post-second-dose) were assessed using generalized estimating equations to account for both within and between patient variability. These models were adjusted for vaccine brand, participant weight and length at month 24, and sex. The statistical analysis was conducted using IBM SPSS Statistics v23.0 (IBM Corp., Armonk, NY, USA), STATA version 17 (STATA Corp, College Station, TX, USA), and GraphPad Prism v9.4.1 (GraphPad, San Diego, CA, USA). A *p*-value of <0.05 was considered statistically significant.

## 3. Results

### 3.1. Demographic Characteristics of Study Participants

**Figure 1:**
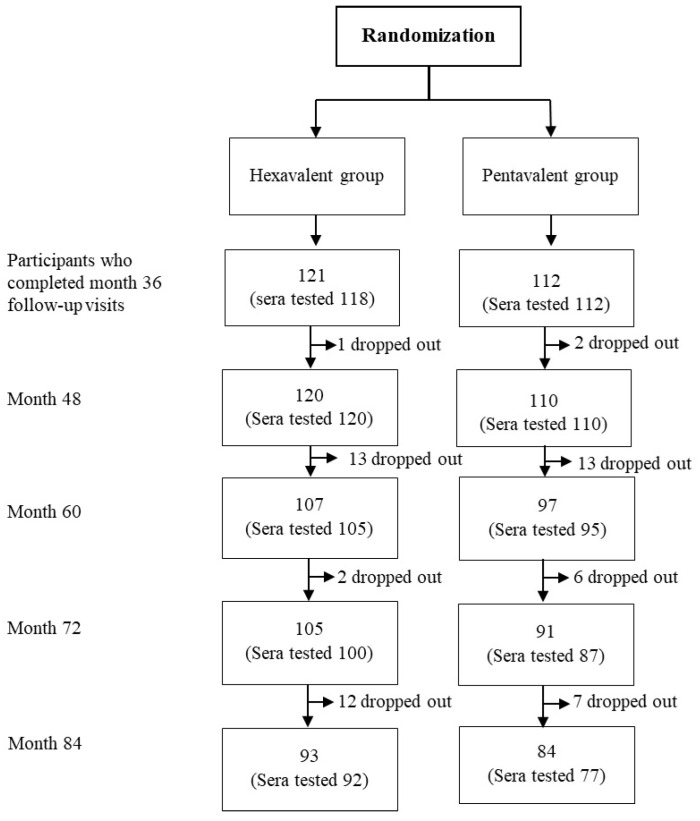
The consort flow diagram of this study.

A total of 230 participants who completed their follow-up visits in month 36 (3 years of age) from the previous study were invited to participate in this extended follow-up study. A total of 93 participants in the hexavalent group and 84 participants in the pentavalent group completed this study at 7 years of age. The total number of sera samples available for testing was 169. The baseline characteristics of the children were previously published in the original study that aimed to evaluate the long-term immunogenicity of pertussis-containing vaccines in this cohort [23]. Briefly, males and females were equally distributed in both hexavalent and pentavalent groups. There were no significant differences in mean weight or height at any of the time points studied. The consort flow diagram of participants is demonstrated in Figure 1. There were 5 children who received M-M-R II for both doses (hereinafter referred to as the MM group), 105 children who received M-M-R II for the first dose and Priorix for the second dose (hereinafter referred to as the MP group), 65 children who received Priorix for the first dose and M-M-R II for the second dose (hereinafter referred to as the PM group), and 53 children who received Priorix for both doses (hereinafter referred to as the PP group).

### 3.2. GMC and Serologic Status of Anti-Measles IgG

**Figure 2:**
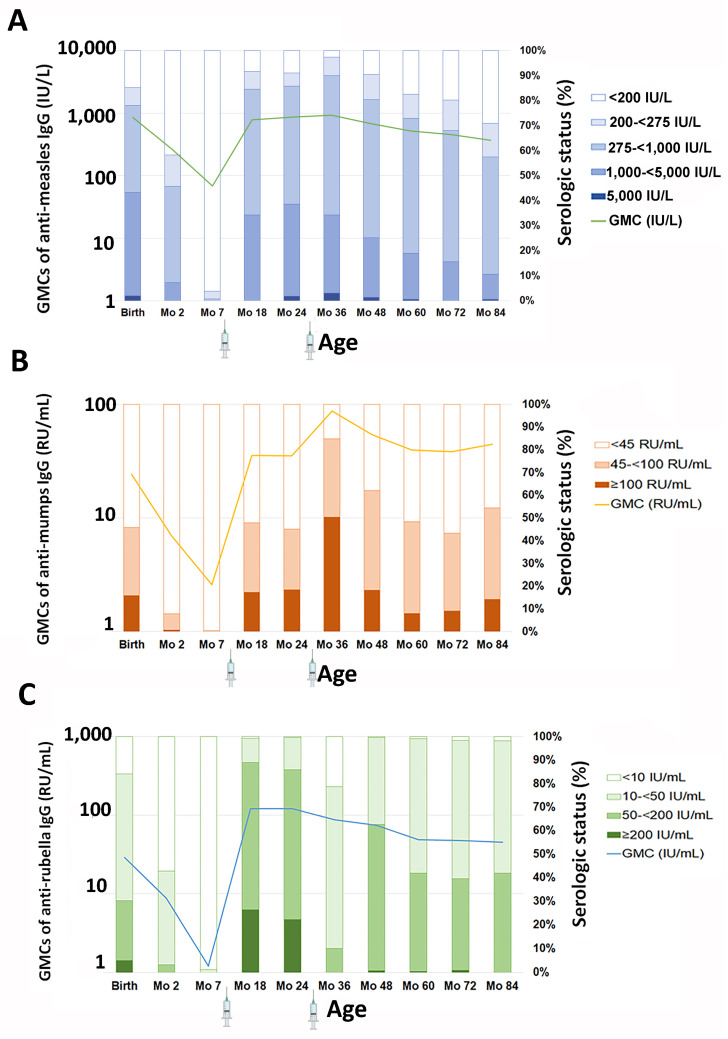
Serologic status and GMCs of antibodies against (**A**) measles, (**B**) mumps, and (**C**) rubella from birth until 7 years of age. The right y-axis represents the percentage of the population with a given antibody concentration; the left y-axis represents the GMC at each age. The syringe icon below the x-axis represents the administration of the MMR vaccine at 9 and 30 months. Data between birth and 36 months were previously published in ref [11].

The GMC of anti-measles IgG decreased from 678.3 IU/L at 4 years to 514.5 IU/L at 5 years, with the GMC further declining to 456.4 and 367.4 IU/L at 6 and 7 years, respectively, as illustrated in Table 1 and Figure 2A. Alongside the GMC reduction, there was a consistent decline in the seroprotection rate with age, with rates of 90.4%, 82.5%, 80.2%, and 71.0% at 4, 5, 6, and 7 years old, respectively, as shown in Table 2. The decay rate of seroprotection was approximately 8% per year since the last-dose vaccination. Looking back at the dynamics since birth, the GMCs generally showed a decline from birth to month 7, reflecting the waning of maternal antibodies. Antibody levels rose following the first vaccination but not significantly following the second vaccination and remained relatively stable over time.

The GMC of anti-measles IgG decreased by 60% at month 84, with a GMC ratio of 0.40 (95% CI 0.37–0.43; *p* < 0.001) compared with month 36. No significant differences were observed in the GMC ratio of anti-measles IgG among participants in the MM, MP, or PM groups compared to those in the PP group. Likewise, there were no differences in anti-measles IgG levels by participant sex, weight, or length (Table 3) at both month 36 and month 84 visits.

### 3.3. GMC and Serologic Status of Anti-Mumps IgG

Similar to anti-measles IgG levels, there was a decline in anti-mumps IgG levels (Figure 2B) after the second-dose vaccination. The GMC of anti-mumps IgG was 54.2 RU/mL at 4 years, 39.7 RU/mL at 5 years, 38.1 RU/mL at 6 years, and 44.5 RU/mL at 7 years of age. Nonetheless, the seropositivity rate for anti-mumps IgG was lower compared with measles. At 4 years of age, approximately 62.2% of the children remained seroprotected. By 5, 6, and 7 years, only 48.5%, 43.3%, and 54.4% of children remained seroprotected, respectively. Regarding the dynamics of anti-mumps IgG since birth, the waning of maternal antibodies between birth and month 7 was similar to that of anti-measles IgG. Antibody levels rose following both the first and second vaccinations but gradually declined over time. By year 7 (month 84), the GMC slightly increased from 38.1 RU/mL to 44.5 RU/mL. None of the children in this study were diagnosed with mumps, suggesting that the rise in antibodies may have been due to natural exposure to the circulating virus.

The GMC of anti-mumps IgG decreased by 49% at month 84, with a GMC ratio of 0.51 (95% CI 0.47–0.56; *p* < 0.001) compared with month 36. No significant differences were observed in the GMC ratio of anti-mumps IgG among participants in the MM, MP, or PM groups compared to those in the PP group. Likewise, there were no differences in anti-mumps IgG levels by participant sex, weight, or length (Table 3) at both month 36 and month 84 visits.

### 3.4. GMC and Serologic Status of Anti-Rubella IgG

As shown in Figure 2C, the GMC of anti-rubella IgG decreased from 73.8 IU/mL at 4 years to 48.7 IU/mL at 5 years. Subsequently, the GMC remained relatively stable at 47.4 IU/mL at 6 years and 44.9 IU/mL at 7 years. Consistently high seroprotection rates (exceeding 98%) against rubella were observed between the ages of 4 and 7 years. No differences in the GMC of anti-measles, -mumps, or -rubella IgG between children who received hexavalent and pentavalent vaccines were observed at any of the tested time points. Anti-rubella IgG also showed waning between birth and month 7. Similar to anti-measles responses, anti-rubella IgG rose following the first vaccination but did not significantly increase after the second vaccination. Although the GMCs gradually declined over time, more than 98% of children remained seroprotected, as defined by anti-rubella IgG levels ≥ 10 IU/mL.

At month 84, the GMC of anti-rubella IgG decreased by 49% compared with month 36, with a GMC ratio of 0.51 (95% CI 0.48–0.55; *p* < 0.001). No significant differences in the GMC ratio of anti-rubella IgG were observed among participants in the MM, MP, or PM groups compared to those in the PP group. While anti-rubella IgG levels did not differ by participant weight or length at month 36 and month 84, female participants had approximately 21% higher GMCs of anti-rubella IgG than male participants (*p* = 0.02) (Table 3).

## 4. Discussion

To the best of our knowledge, this was the first study in Thailand to evaluate the long-term immunogenicity of the two-dose MMR vaccine administered at 9 months and 2.5 years, with a follow-up 4.5 years post-vaccination (7 years of age). We found that the GMCs of anti-measles, anti-mumps IgG, and anti-rubella IgG declined over time, while the seropositivity rate declined for measles and mumps but not for rubella. The seroprotection rate against measles observed in this study (range 71–90% in children aged 4–7 years) closely resembled that of a previous report on Thai children, ranging from 85.3% in children aged 3–5 years to 72.5% in children aged 6–9 years [24]. The previous Thai study also reported a further decline in anti-measles IgG levels and seroprotection rates among adolescents who had previously received the two-dose MMR vaccine [24]. Over a span of 12 years, a cohort study in the USA monitored the vaccine-induced immune response in individuals who received the two-dose MMR vaccine at 12–15 months and at 4–6 years. The GMC of anti-measles antibodies decreased by an average of 7.4–9.7% annually in that cohort [25], lower than the decay rate of 60% between 3 and 7 years observed in the current study. Individuals immunized with two doses of the measles vaccine might not maintain a protective antibody titer several years after vaccination [26]. Nonetheless, the decline in circulating antibodies does not necessarily equate to loss of protection as evidenced by a study reporting that 93% retained immunological memory for measles after receiving a first or second booster dose [26]. In addition, the effectiveness of two doses of MCV remains high over decades post-immunization, with only slight degradation [27]. These results underscore the critical need to achieve high vaccination coverage with two doses of MCV to prevent measles outbreaks in the community.

Our findings indicate a decline in the seroprotection rate for mumps after 4.5 years post-two-dose MMR vaccination, with rates dropping to 54% by the age of 7 years. A previous study conducted in Thailand in 2020 noted a mumps seropositivity rate of 82.1%, determined using a lower cut-off of 22 RU/mL, in children between 3 and 9 years of age [28]. The seroprotection rate in that study further declined to 41.7% in adolescents [28]. The rapid waning of mumps immunity could be due to the lack of exposure to a natural booster, as Thailand has not experienced a mumps outbreak for many years. The seroprotection rates, ranging from 43 to 62% in this study, fall below the estimated threshold for herd immunity of 84–86% [29]. Therefore, if an outbreak were to occur, Thai children would be susceptible to mumps. In 2017, the Advisory Committee on Immunization Practices (ACIP) recommended a third dose of a mumps-virus-containing vaccine for persons previously vaccinated with two doses who had been identified by public health authorities as being part of a group at increased risk for acquiring mumps because of an outbreak [30]. This third-dose vaccine, along with other public health measures, e.g., the isolation of infectious persons, timely contact tracing, and effective communication and awareness education for the public and medical community, should remain key interventions for outbreak control [30].

In contrast, rubella seroprotection rates remained consistently high throughout the study period, exceeding 98% from ages 4 to 7 years. This level of protection surpasses the herd immunity threshold of 85% to 88% [19]. A prior study conducted in Thailand in 2020 reported a similar range of rubella seroprotection rates at 97.0% among children aged 3 to 9 years who had received two doses of the MMR vaccine [20]. A longitudinal 12-year follow-up study on rubella antibody persistence also reported a faster decline for anti-measles and anti-mumps compared with anti-rubella antibodies [25]. This robust and sustained protection against rubella reaffirms the reliability and durability of rubella immunity within the Thai population.

Our study had some limitations. First, enzyme immunoassay for virus-specific IgG measures antibodies to abundant viral proteins and has limited ability to measure antibodies to conformational epitopes; thus, their utility for categorization of individuals as immune or non-immune is limited. Second, this study did not evaluate the cellular immunity or other parameters that may be associated with durable and/or protective immunity after vaccination [31]. In addition, Thailand experienced an increase in the incidence of measles and mumps between 2018 and 2019, overlapping with the study period [32,33]. Thus, children in this study may have been exposed to circulating viruses. This exposure could result in increased antibody levels, as evidenced by the slight increases in anti-mumps IgG observed at 7 years of age in this cohort. Another limitation of our study was the absence of an established antibody correlate of protection for mumps. The chosen cut-off of 45 RU/mL for anti-mumps IgG was arbitrary and necessitated careful interpretation of the data. Future research should focus on establishing a validated correlate of protection for mumps to enhance the accuracy and reliability of immunogenicity assessments in both clinical and public health contexts.

Although the seroepidemiological study is a potentially powerful tool for monitoring the durability of immune responses following vaccination programs, the waning of vaccine-induced antibodies may not correlate with a lack of immunity. Further studies including functional assays, e.g., antibody avidity or neutralizing activity, across diverse populations vaccinated with varying vaccine schedules are warranted and may contribute to the understanding of MMR vaccine-induced protection.

## 5. Conclusions

In conclusion, our study findings expanded the current understanding of long-term antibody persistence to measles, mumps, and rubella viruses after the two-dose MMR vaccination scheduled at 9 months and 2.5 years, as recommended by the national immunization program of Thailand. We reported a decline in seroprotection rate against measles and mumps, but not rubella. Longitudinal monitoring of antibody persistence, among other strategies, could help predict population-level immunity and inform public health interventions to address potential future outbreaks.

## Figures and Tables

**Table 1:** Geometric mean concentrations (GMCs) and 95% confidence intervals (CIs) of anti-measles IgG, anti-mumps IgG, and anti-rubella IgG at different ages in children who received hexavalent and pentavalent vaccines.

Time Points	Antibody	Overall GMCs (95% CI)	GMCs of Children Receiving Hexavalent Vaccine (95% CI)	GMCs of Children Receiving Pentavalent Vaccine (95% CI)
Month 48	Anti-measles IgG (IU/L)	678.3 (598.8–768.3)	716.1 (600.1–854.5)	639.3 (536.3–762.1)
	Anti-mumps IgG (RU/mL)	54.2 (49.5–59.4)	58.8 (52.4–65.9)	49.6 (43.0–57.2)
	Anti-rubella IgG (IU/mL)	73.8 (67.9–80.2)	77.6 (69.6–86.5)	69.8 (61.5–79.3)
	n	230	120	110
Month 60	Anti-measles IgG (IU/L)	514.5 (445.8–593.9)	532.1 (437.9–646.4)	495.8 (400.8–613.3)
	Anti-mumps IgG (RU/mL)	39.7 (35.8–44.0)	44.9 (39.3–51.3)	34.6 (29.5–40.5)
	Anti-rubella IgG (IU/mL)	48.7 (44.2–53.6)	52.3 (45.9–59.6)	45.0 (39.1–51.8)
	n	200	105	95
Month 72	Anti-measles IgG (IU/L)	456.4 (397.6–523.9)	450.7 (374.3–542.7)	463.2 (376.7–569.5)
	Anti-mumps IgG (RU/mL)	38.1 (34.0–42.8)	43.4 (37.2–50.6)	32.9 (27.8–38.8)
	Anti-rubella IgG (IU/mL)	47.4 (42.8–52.6)	49.7 (43.5–56.7)	44.9 (38.2–52.9)
	n	187	100	87
Month 84	Anti-measles IgG (IU/L)	367.4 (313.9–430.1)	360.5 (291.8–445.3)	375.9 (296.4–476.6)
	Anti-mumps IgG (RU/mL)	44.5 (39.7–49.9)	48.2 (41.7–55.8)	40.5 (33.9–48.4)
	Anti-rubella IgG (IU/mL)	44.9 (40.6–49.7)	46.8 (41.0–53.3)	42.8 (36.6–50.2)
	n	169	92	77

**Table 2:** Percentages of children with different levels of anti-measles, anti-mumps, and anti-rubella IgG between 4 and 7 years of age (48 to 72 months) with 95% confidence intervals.

	48 Months	60 Months	72 Months	84 Months
Anti-measles IgG				
<200 IU/L	9.6% (5.8–13.4)	17.5% (12.2–22.8)	19.8% (14.1–25.5)	29.0% (22.2–35.8)
200–<275 IU/L	10.0% (6.1–13.9)	9.5% (5.4–13.6)	12.3% (7.6–17.0)	13.6% (8.4–18.8)
275–<1000 IU/L	55.2% (48.8–61.6)	54.0% (47.1–60.9)	52.4% (45.2–59.6)	46.7% (39.2–54.3)
1000–<5000 IU/L	23.9% (18.4–29.4)	18.5% (13.1–23.9)	15.5% (10.3–20.7)	10.1% (5.5–14.6)
≥5000 IU/L	1.3% (−0.2–2.8)	0.5% (−0.5–1.5)	0.0% (0.0–0.0)	0.6% (−0.6–1.7)
Anti-mumps IgG				
<45 RU/mL	37.8% (31.6–44.1)	51.5% (44.6–58.4)	56.7% (49.6–63.8)	45.6% (38.1–53.1)
45–<100 RU/mL	43.9% (37.5–50.3)	40.5% (33.7–47.3)	34.2% (27.4–41.0)	40.2% (32.8–47.6)
≥100 RU/mL	18.3% (13.3–23.3)	8.0% (4.2–11.8)	9.1% (5.0–13.2)	14.2% (8.9–19.5)
Anti-rubella IgG				
<10 IU/mL	0.4%(−0.4–1.3)	1.0% (−0.4–2.4)	1.6% (−0.2–3.4)	1.8% (−0.2–3.8)
10–<50 IU/mL	37.0% (30.7–43.2)	57.0% (50.1–63.9)	58.8% (51.8–65.9)	56.2% (48.7–63.7)
50–<200 IU/mL	61.7% (55.5–68.0)	41.5% (34.7–48.3)	38.5% (31.5–45.5)	42.0%(34.6–49.5)
≥200 IU/mL	0.9% (−0.3–2.1)	0.5% (−0.5–1.5)	1.1% (−0.4–2.5)	0.0% (0.0–0.0)

**Table 3:** Multivariable model showing geometric mean concentration (GMC) ratios of anti-measles, anti-mumps, and anti-rubella IgG.

	GMC Ratios	95% CI	*p*-Value
Anti-measles IgG			
Study month			
36	1 (ref)		
84	0.40	(0.37–0.43)	<0.001
Vaccine type			
MM	1.33	(0.57–3.12)	0.51
MP	0.98	(0.72–1.35)	0.92
PM	0.97	(0.69–1.35)	0.85
PP	1 (ref)		
Weight at month 24 (kg)	0.99	(0.91–1.09)	0.93
Length at month 24 (cm)	0.98	(0.93–1.02)	0.32
Sex			
Male	1 (ref)		
Female	0.99	(0.78–1.28)	0.99
Anti-mumps IgG			
Study month			
36	1 (ref)		
84	0.51	(0.47– 0.56)	<0.001
Vaccine type			
MM	1.06	(0.59–1.91)	0.85
MP	0.98	(0.79–1.21)	0.84
PM	1.02	(0.81–1.29)	0.85
PP	1 (ref)		
Weight at month 24 (kg)	1.01	(0.95–1.07)	0.85
Length at month 24 (cm)	0.99	(0.96–1.03)	0.74
Sex			
Male	1 (ref)		
Female	1.09	(0.92–1.29)	0.31
Anti-rubella IgG			
Study month			
36	1 (ref)		
84	0.51	(0.48–0.55)	<0.001
Vaccine type			
MM	1.50	(0.86–2.61)	0.16
MP	1.05	(0.86–1.29)	0.61
PM	1.06	(0.85–1.32)	0.58
PP	1 (ref)		
Weight at month 24 (kg)	0.99	(0.93–1.05)	0.75
Length at month 24 (cm)	0.99	(0.96–1.02)	0.42
Sex			
Male	1 (ref)		
Female	1.21	(1.03–1.42)	0.02

CI, confidence interval; MM, participants who received M-M-R II for both doses; MP, participants who received M-M-R II for the first dose and Priorix for the second dose; PM, participants who received Priorix for the first dose and M-M-R II for the second dose; PP, participants who received Priorix for both doses; kg, kilogram; cm, centimeter.

## Data Availability

All data are available in this manuscript. Additional information can be obtained from the corresponding authors upon reasonable request.
